# A Case of Systemic Mastocytosis Diagnosed Endoscopically

**DOI:** 10.7759/cureus.32329

**Published:** 2022-12-08

**Authors:** Ariana R Tagliaferri, Devina Adalja, Yana Cavanagh

**Affiliations:** 1 Internal Medicine, St. Joseph’s Regional Medical Center, Paterson, USA; 2 Internal Medicine, St. Joseph’s University Medical Center, Paterson, USA; 3 Gastroenterology, St. Joseph’s Regional Medical Center, Paterson, USA

**Keywords:** tryptase, mucosal nodularity, abdominal pain, endoscopic biopsy, urticaria, cutaneous mastocytosis, systemic mastocytosis

## Abstract

Mastocytosis, or mast cell proliferation, is very rare. Overall, 60% of patients with systemic mastocytosis (SM) have gastrointestinal involvement, with the colon being the most prevalent affected site. Most patients are diagnosed by bone marrow biopsy. Although gastrointestinal symptoms are common, patients are rarely diagnosed via endoscopy. Indolent SM, which is characterized by both gastrointestinal and cutaneous symptoms in the absence of bone marrow suppression, is extremely rare and often missed due to the complexity of the diagnosis. Here, we present the case of a patient with abdominal pain, flushing, and nausea who was diagnosed endoscopically with SM, likely the indolent type.

## Introduction

Mast cell progenitors migrate from the bone marrow and mature in well-vascularized tissues, such as the nerves, smooth muscles, and glandular tissue [1]. They are activated when exposed to certain antigens and release histamine, cytokines, leukotrienes, and proteases, causing a variety of effects in their respective tissues [1]. Mastocytosis, or mast cell proliferation, is an uncommon disorder of the immune system, with an estimated incidence of two cases per 100,000 each year [1,2]. Mastocytosis was first described in France in 1930; however, the first instance of urticaria as an effect of mast cell activation traces back to 1869 [3]. Although it affects both men and women alike, the systemic form is more prevalent in adults compared to children [1]. Mast cell overactivity is a result of a somatic mutation in the *KIT *gene in the 816th codon where valine is substituted for an aspartate [3,4]. This gain-of-function mutation is positive in more than 90% of patients with systemic mastocytosis (SM) [4].

The 2008 World Health Organization Classification defines mastocytosis as either cutaneous mastocytosis (CM) or SM, although subdivisions of these main classes do exist [1]. More than 90% of all mastocytosis is CM, in which patients may present with a constellation of urticaria, flushing, or maculopapular rashes [3]. Specifically, these rashes occur on the torso and extremities but spare the palms and soles [3]. This can only be considered CM if biopsies from other organs are negative [3]. Secondary to cutaneous symptoms, the next most common site of involvement is bone marrow involvement, which manifests as pancytopenia and/or lytic bone fractures [1-3]. Other sites of SM include the gastrointestinal tract, lymphatic tissue, liver, spleen, and urogenital system causing symptoms of abdominal pain, nausea, vomiting, elevated liver enzymes, malabsorption, and hypoalbuminemia [1,2]. Indolent SM is diagnosed when there are gastrointestinal and cutaneous symptoms but without end-organ damage and bone marrow involvement [3]. This subtype is extremely rare.

To diagnose either subtype, one must fulfill one major and one minor criterion, or fulfill at least three minor criteria [1]. In general, the diagnoses must include typical clinical symptoms with an increase in total serum tryptase greater than 20% from baseline, and/or an improvement in symptoms following the administration of mast cell-blocking drugs [3]. A biopsy is confirmatory and gold standard [1,3]. As indolent SM is extremely rare, the majority of patients are diagnosed via bone marrow biopsy [2]. Here, we present the case of a 39-year-old female who presented with abdominal pain, flushing, and nausea and was diagnosed endoscopically with SM, likely the indolent type [2].

This case report was previously presented at the ACG Annual Scientific Meeting 2022 as a Poster Presentation and published in the American Journal of Gastroenterology as an abstract. The same authors have approved this case report for full manuscript publication.

## Case presentation

A 39-year-old female with no significant medical history presented to the Gastroenterology Clinic for evaluation of a peri-appendiceal mass. The patient was initially seen by her primary care provider for dull and non-radiating right lower quadrant pain, associated with extreme flushing of her face, lips, ears, chills, and nausea. A review of systems was otherwise negative. She was referred to an allergist, at which time tryptase was positive. To evaluate the abdominal pain, a computerized tomography (CT) scan of the abdomen and pelvis without contrast was performed, which showed a soft-tissue nodule along the right lower quadrant near the ileocecal region measuring 1.5 × 1.2 cm. There were two mesenteric lymph nodes measuring 1 × 1.4 cm and 1 × 0.7 cm. She reportedly underwent a positron emission tomography (PET) scan which was negative; however, imaging could not be obtained, as this was all performed at an out-of-state hospital.

When she was referred to the Gastroenterology Clinic, her blood pressure was 103/65 mm/Hg, heart rate was 126 beats per minute, respiratory rate was 18 breaths per minute, and she was saturating 100% on room air. She was alert, well-nourished with moist mucous membranes, had no appreciable lymphadenopathy, her abdomen was soft and non-tender to palpation and non-distended, and had normoactive bowel sounds without appreciable organomegaly or abdominal masses. The perianal and digital rectal examinations were normal, and the patient demonstrated normal sphincter tone. Laboratory studies were significant for normocytic anemia (hemoglobin 11.7 g/dL, mean corpuscular volume 90.6 fL); however, she had normal complement levels and negative inflammatory markers (C-reactive protein and erythrocyte sedimentation rate), and there were no electrolyte abnormalities or leukocytosis.

**Figure 1:**
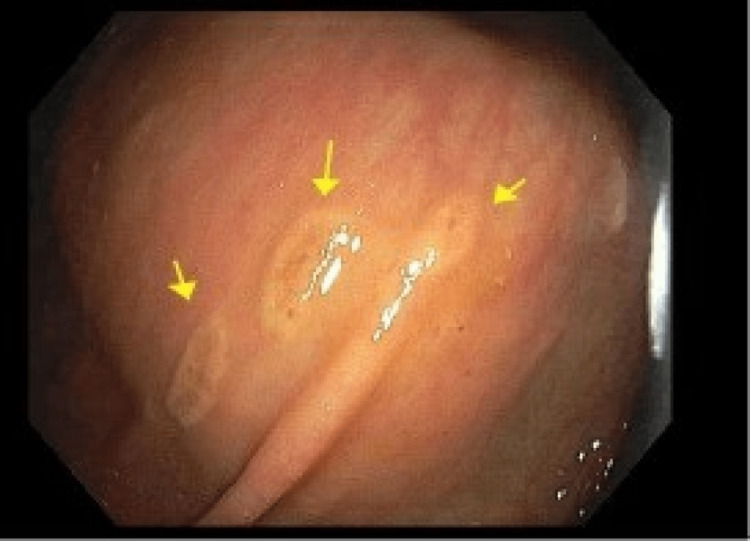
Macroscopic evidence of mastocytosis on esophagogastroduodenoscopy. Arrows point to yellowish-white mucosal nodules with central hyperpigmentation visualized in the descending colon.

**Figure 2:**
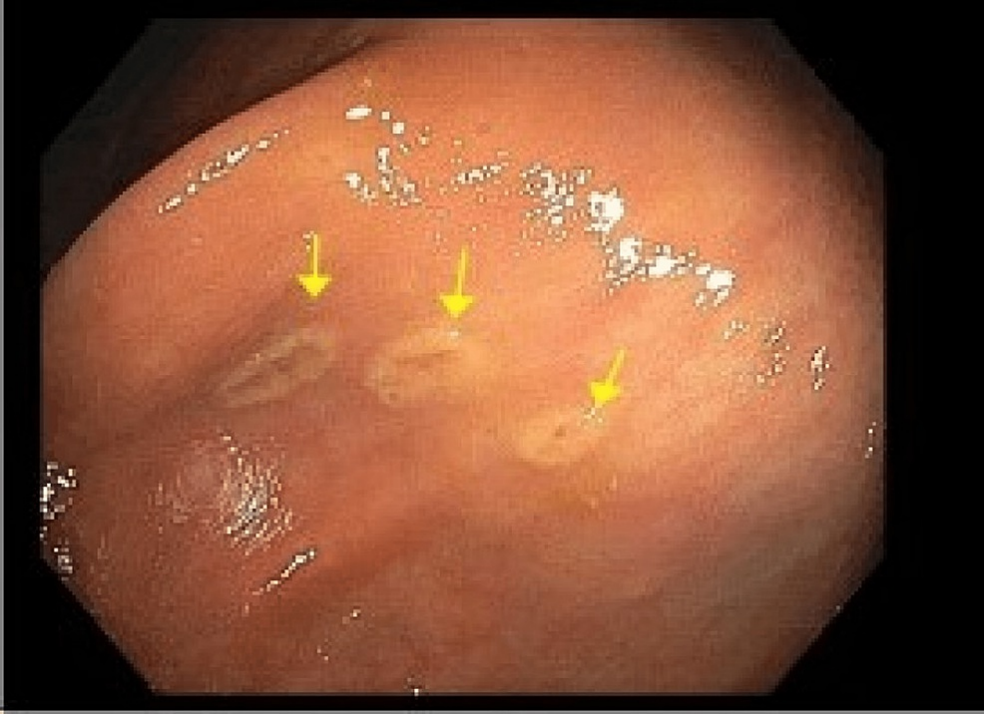
Macroscopic evidence of mastocytosis on esophagogastroduodenoscopy in the transverse colon. Arrows indicate yellowish-white mucosal nodules with central hyperpigmentation visualized in the transverse colon.

Initially, a carcinoid tumor was suspected and the patient underwent a colonoscopy with endoscopic ultrasound (EUS) and biopsy. There was no bleeding, and grade I internal hemorrhoids and three sessile polyps were found in the rectum (6 mm and 10 mm) and transverse colon (22 mm). They were biopsied with pathology and confirmed to be tubulo-villous adenomas. Many 5-15 mm yellow-white mucosal nodules with central hyperpigmentation were found in the entire colon, as well as discontinuous areas of non-bleeding and ulcerated mucosa in the transverse colon, ascending colon, and cecum (Figures 1, 2).

**Figure 3:**
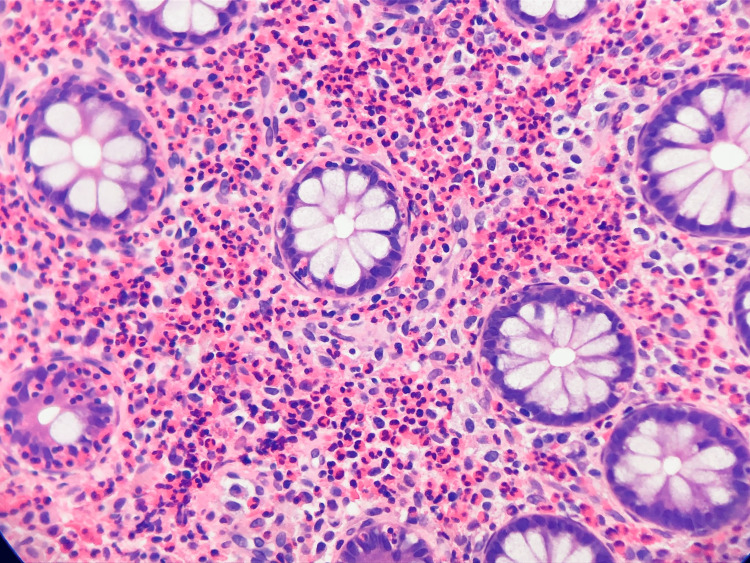
Microscopic evidence of mastocytosis on hematoxylin and eosin stain. Hematoxylin and eosin stain showing eosinophils and mast cell proliferation within the cryptic architecture.

**Figure 4:**
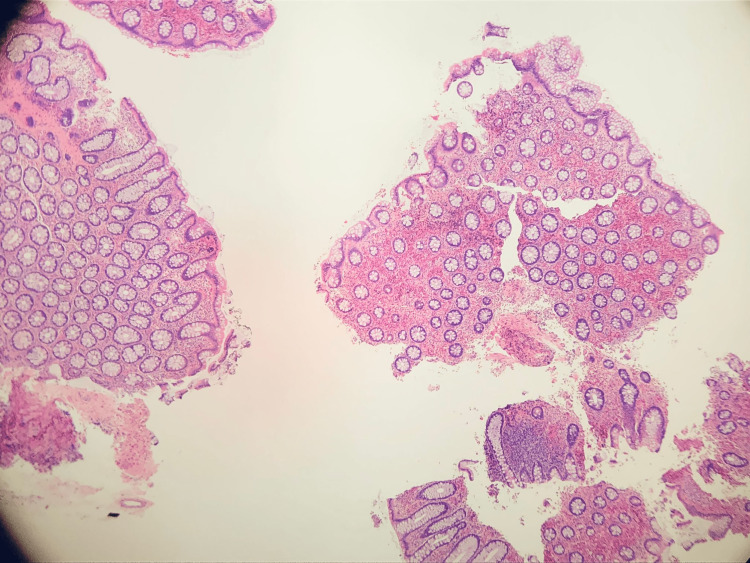
Hematoxylin and eosin stain from the Ileocecal mucosa. Ileocecal mucosa with markedly increased eosinophils and abnormal mast cell proliferation in the lamina propria.

Cold forceps biopsy was performed, and samples were sent to histology. The terminal ileum appeared macroscopically normal. Endoscopic imaging showed no remarkable pathology. The final pathology report revealed colonic mucosa with markedly increased eosinophils and abnormal mast cell proliferation in the lamina propria from the right colonic mucosal plaques, ileocecal mucosa, and ileocecal valve samples. There was ileal mucosa with focal small clusters of abnormal mast cells in the lamina propria and colonic mucosa with focal erosion in the right colon in general (Figures 3, 4).

**Figure 5:**
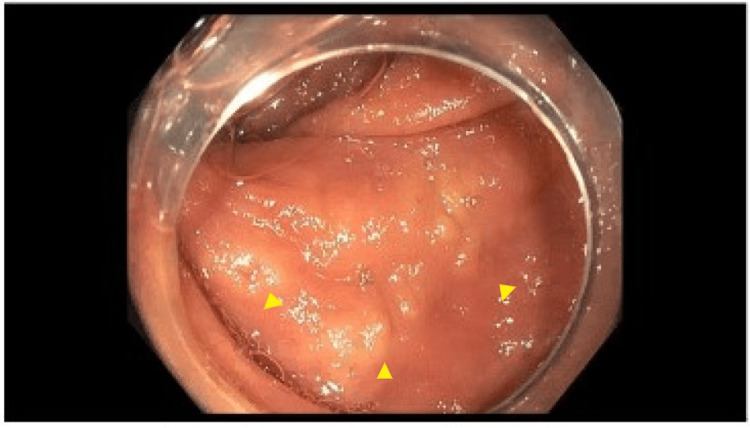
Surveillance colonoscopy with irregular mucosa. Arrows show irregular patchy and nodular mucosa of the ascending colon.

**Figure 6:**
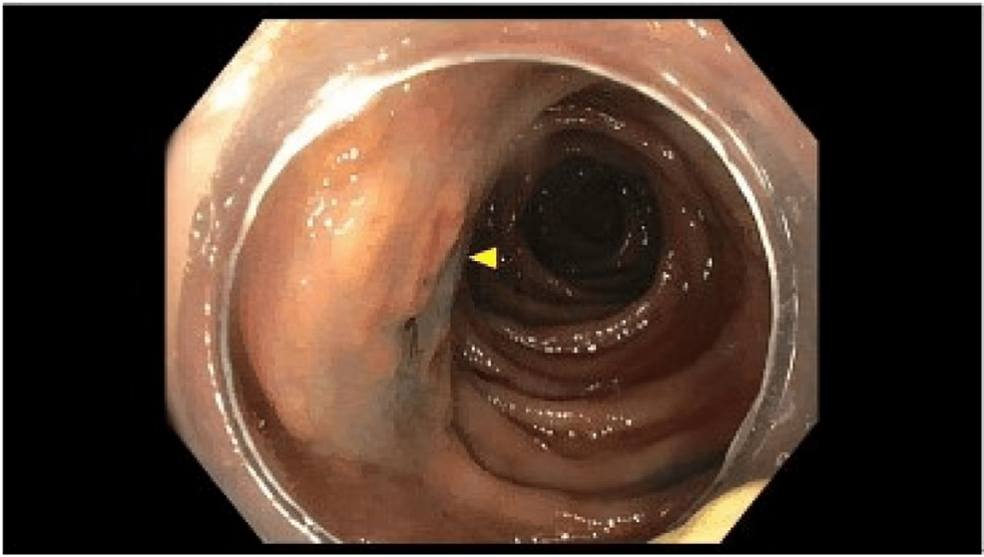
Surveillance colonoscopy with recurrent mastocytosis. The arrow reflects the nodularity of the ascending colon.

**Figure 7:**
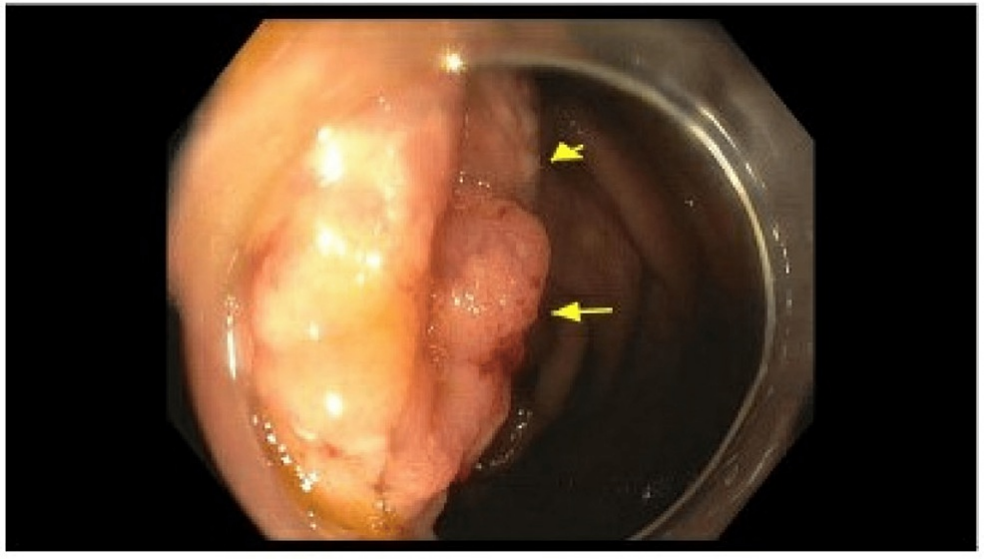
Surveillance colonoscopy with mucosal nodularity. Arrows demonstrate the nodularity of the ileocecal valve.

The ileal and colonic mucosa showed sheets of eosinophils mixed with clusters of mast cells, showing atypical morphology, including oval-to-short, spindled nuclei and focal clustering. Immunohistochemical stains showed atypical mast cells positive for CD117, CD25, and tryptase (subset) and negative for CD2 and S100. OnkoSight NGS KIT sequencing detected Tier 1 genomic alterations in *KIT p.Asp816Val*, which strongly supported the clinical diagnosis of SM. She was started on loratadine and famotidine. A repeat colonoscopy was performed, indicative of persistent disease (Figures 5-7).

She was referred to Rheumatology for aggressive therapy.

## Discussion

Only 10% of all mastocytosis is considered systemic, with the most common site of involvement being the bone marrow [2,5]. However, of those patients with SM, approximately 60% also have gastrointestinal symptoms [2,5]. A case series observing the clinicopathologic features in five patients determined the colon to be the most prevalent site of involvement [2,5]. Four out of five patients had cutaneous symptoms, gastrointestinal symptoms, and bone marrow involvement; however, up to 50% of patients with SM lacked cutaneous symptoms at the time of diagnosis [5]. Endoscopically, patients were found to have mucosal nodularity, loss of normal architecture, and friability with pathologically positive dense mast cell infiltration of the lamina propria [2,5]. This is similar to our patient who had colonic involvement characterized by mucosal nodularity, friability, and ulceration; however, our patient likely had indolent SM given the absence of bone marrow suppression. Our patient was also unique in the way she was diagnosed endoscopically. Most patients present with characteristic urticaria, flushing, and a constellation of laboratory findings leading them to ultimately undergo skin and/or bone marrow biopsies [4]. Not only are less than 10% of patients found to have bone marrow involvement warranting biopsy but fewer have isolated gastrointestinal symptoms [3,4]. There are no current studies to illustrate the incidence of diagnosis via endoscopic measures. It is important to rule out alternative diagnoses such as celiac disease, irritable bowel syndrome, fibromyalgia, carcinoid tumors, pheochromocytomas, or vasculitis [3]. Serum tryptase can be helpful but has limitations as it does not correspond to the SM subtype or disease burden [4]. For example, serum tryptase may be normal when bone marrow involvement is absent, as in the case of our patient [4].

Treatment and prognosis are determined based on the subtype and genetic analysis [3]. Treatment focuses on reducing symptoms but is not curative [1,3]. Given the clinical heterogeneity, the prognosis also subsequently varies [1,3]. One study illustrated how individuals with SM often carry secondary mutations associated with myeloid neoplasms, such as *JAK2 V617F*, *TP53*, *SRSF2*, *ASXL1*, *RUNX1*, or *TET2 *[3]. Unfortunately, for those with the *KIT D816V* mutation, their disease is resistant to tyrosine kinase inhibitors such as imatinib and masitinib [4]. Treatment otherwise includes anti-mast cell agents, steroids, and anti-histamines but guidelines are poor [1].

## Conclusions

Mastocytosis is a rare disease characterized by heterogeneous clinicopathological features and variable treatment and prognosis. In the absence of classical cutaneous lesions, bone marrow suppression, and/or serum tryptase elevation, the diagnosis of indolent SM can be easily missed. As many patients lack cutaneous symptoms at the time of diagnosis, clinical suspicion should remain high if other more common diseases can be excluded. This is a rare case of indolent SM diagnosed endoscopically.
